# Effect of Cryostructuring Treatment on Some Properties of Xanthan and Karaya Cryogels for Food Applications

**DOI:** 10.3390/molecules26092788

**Published:** 2021-05-09

**Authors:** Jonathan Coria-Hernández, Rosalía Meléndez-Pérez, Abraham Méndez-Albores, José Luis Arjona-Román

**Affiliations:** 1 Laboratory 13 Thermal and Structural Analysis of Materials and Foods, Multidisciplinary Research Unit, Superior Studies Faculty at Cuautitlan, National Autonomous University of Mexico, Cuautitlan Izcalli 54714, Mexico; melendez@unam.mx (R.M.-P.); arjona@unam.mx (J.L.A.-R.); 2 Laboratory 14 A-1 Materials Science and Technology, Multidisciplinary Research Unit, Superior Studies Faculty at Cuautitlan, National Autonomous University of Mexico, Cuautitlan Izcalli 54714, Mexico; albores@unam.mx

**Keywords:** xanthan gum, karaya gum, hydrogels, cryostructuring, cryogels

## Abstract

Cryogels are novel materials because the manufacturing process known as cryostructuring allows biopolymers to change their properties as a result of repeated controlled freeze–thaw cycles. Hydrogels of xanthan and karaya gums were evaluated after undergoing up to four controlled freeze–thaw cycles in indirect contact with liquid nitrogen (up to −150 °C) to form cryogels. Changes in structural, molecular, rheological, and thermal properties were evaluated and compared to those of their respective hydrogels. Samples were also analyzed by Scanning Electron Microscopy (SEM), Fourier Transform Infrared Spectroscopy with Attenuated Total Reflection (FTIR-ATR), Rotational Rheology (RR), Modulated Differential Scanning Calorimetry (MDSC) and zeta potential (ζ). In general, significant differences (*p* < 0.05) between the numbers of freeze–thaw cycles were found. Karaya cryogels were not stable to repeated cycles of cryostructuring such as the three-cycle xanthan cryogel, which has the best structural order (95.55%), molecular interactions, and thermal stability, which allows the generation of a novel material with improved thermal and structural properties that can be used as an alternative in food preservation.

## 1. Introduction

**Figure 1:**
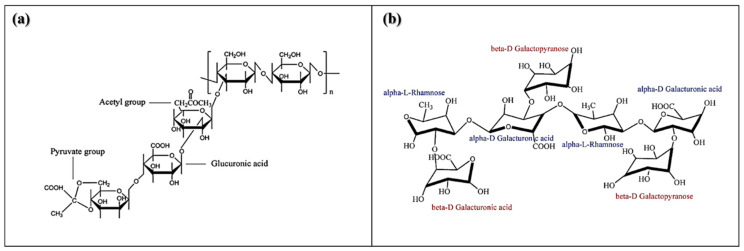
Chemical structures of (**a**) xanthan gum and (**b**) karaya gum [4,5].

Xanthan gum is a branched heteropolysaccharide synthesized by different species of bacteria, among them *Xanthomonas campestris*, which produces the polymer as a protection mechanism. It is formed by residues of D-glucose, D-mannose and D-glucuronic acid (Figure 1a). Xanthan also contains approximately 4.7% of acetyl groups and 3.5% pyruvic acid, and its molecular weight is approximately 3000 kDa. Xanthan is a polysaccharide that forms high viscosity and stable pseudoplastic dispersions over a variety of pH values, as well as in the presence of various salts. Xanthan is mostly soluble in cold water, is partially resistant to enzymatic degradation, functions as a good cryoprotectant, and is compatible with other gums, presenting greater synergy with galactomannans [1].

In order to get better functional properties, xanthan must be properly hydrated, which depends on four important factors: (1) dispersion mechanism, (2) solvent agitation speed, (3) chemical composition of the solvent, and (4) particle size.

Current knowledge on the structure of xanthan hydrogels explains many of its properties. However, its behavior results mainly from the ability of dispersed molecules to form inter- and intra-molecular aggregates [2,3].

On the other hand, karaya gum is a plant exudate of the *Sterculia urens* family. This polysaccharide was introduced as a substitute for tragacanth gum, and due to its nature, it has many uses around the world. In general, karaya gum is used in industry as a stabilizer and adhesive. Karaya gum is partially acetylated at approximately 8%, has approximately 37% uronic acid (Figure 1b), an equivalent weight of 511 kDa, and a molecular weight of approximately 9500 kDa [6].

Although karaya is classified as a gum easily dispersible in water, this polysaccharide possesses lower solubility. The particles in the water swell, giving them their particular characteristic, since the rheological behavior depends mainly on the type of dispersion and the size of the particles.

Cryogels produced from natural polymers, unlike synthetic polymers, significantly change their degree of structural order by undergoing minor modifications in factors such as temperature, pH, or ionic forces. The reorientation of cryogels favors the formation of inter- and intra-molecular bonds between polymer–polymer and/or polymer–water and the number of these bonds can vary widely, changing their properties [7,8]. The processes to prepare this type of material are known as “cryogelation” and “cryostructuring”. This means that the changes produced in the materials are mainly induced by the cryogenic influence, including liquid to solid phase transitions of the solvent that interacts with the polymer. Two types of materials can be obtained from these processes: (1) macroporous matrices ranging from 0.1 to 10 µm, and (2) super macroporous matrices with sizes greater than 10 µm. Furthermore, the shape and size of the pores in these materials depend mainly on the nature of the polymer, concentration, the solvent used, and the freeze–thaw conditions, such as rate and time [8,9,10,11,12].

The study of cryogels has potential applications in various areas such as medicine, bioengineering, environment, and others. Although there is no concrete research yet, their use in food engineering can be very useful to assist conservation processes [8,11].

In both cases, it is important to note that both xanthan and karaya gum are widely used in the food industry and are *Generally Recognized As Safe* (GRAS) substances with various properties, including increased viscosity, improved stability of dispersed systems, gelling properties, water holding retention, and texture improvement. Its applications include beverages, dairy, bakery and meat products.

The objective of this work was to evaluate the thermal, molecular, rheological, and structural behavior of xanthan and karaya hydro- and cryogels submitted with up to four freeze–thaw cycles to produce a material with novel properties for possible applications in the food preservation/transformation industry.

## 2. Results and Discussion

### 2.1. SEM Analysis

**Figure 2:**
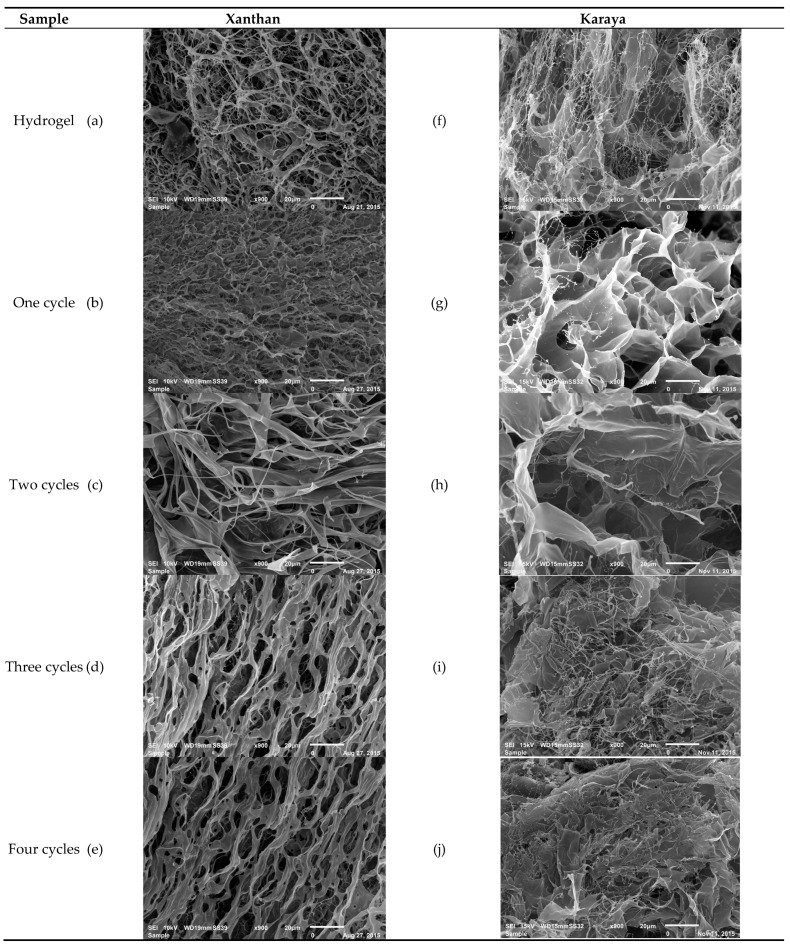
SEM micrographs (900×) of the xanthan and karaya hydro- and cryogels.

Figure 2a–e shows the microstructure of the hydro- and cryogels as a function of the number of cryostructuring cycles, respectively. For xanthan samples, the size of the empty spaces was modified from the first freezing cycle, resulting in more homogeneous pores of smaller size. However, in the second cycle, the spaces increased in size, and also an increment in the heterogeneity was observed. These results agree with those reported by Giannouli and Morris [13].

**Figure 3:**
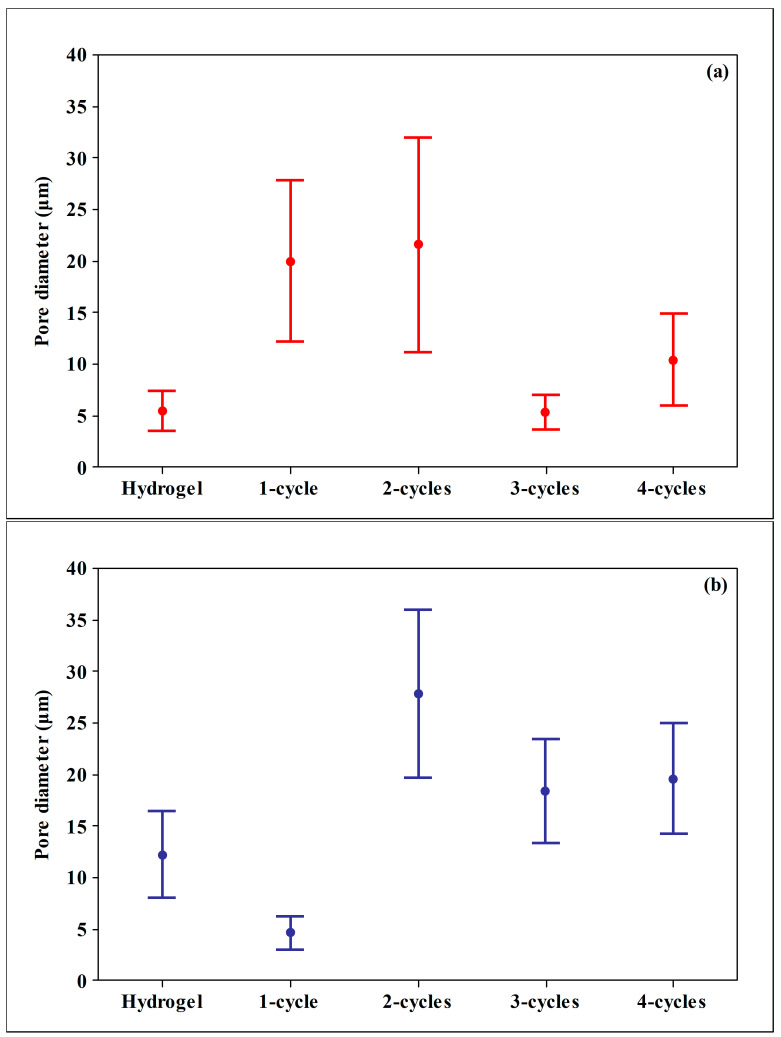
Confidence intervals at 95% of the polymer pore diameter: (**a**) xanthan; (**b**) karaya.

At the third cycle, the structure of the xanthan cryogel was not modified significantly (*p* > 0.05), which may be due to the formation of larger molecular assemblies, mainly of union or dimerization crisscrossed (formation of a double coaxial helix), which promoted the rearrangement of the polymeric chains reaching their highest degree of conformational ordering. Thus, more homogeneous pores were observed (Figure 3a).

Figure 2f–j shows the microstructure of karaya samples subjected to freeze–thaw cycles. In these samples, a high level of heterogeneity between pore diameters was observed since the first cryogel cycle. This phenomenon is related to the structural order degree, since the hydrogel is the one with the smallest pore size and largest arrangement, and when the polymer undergoes cryostructuring, the amorphous part increases, modifying the size and pore distribution (Figure 3b), providing less physicochemical stability.

### 2.2. MDSC Studies

**Figure 4:**
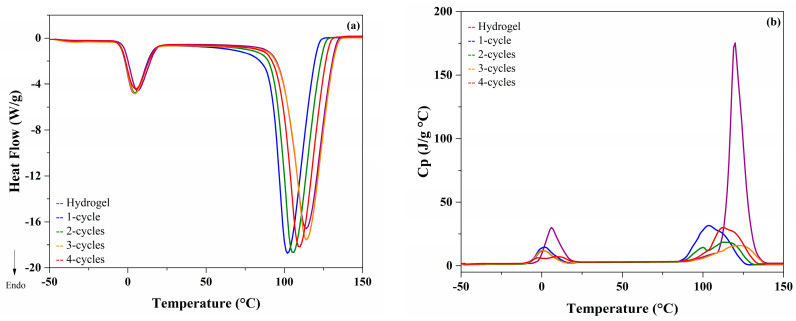
Thermograms of xanthan samples (**a**) Heat flow; (**b**) Specific heat (Cp).

The thermal analysis of the xanthan cryogels (Figure 4a) shows that the first transition corresponds to ice melting. No significant differences (*p* > 0.05) in the total heat flow were recorded. The melting temperature (T_m_) was found between 4 and 6 °C for all samples, regardless of the number of freezing cycles.

The second important transition corresponds to water evaporation. Significant differences (*p* < 0.05) depending on the number of freeze–thaw cycles were observed. In those samples, evaporation temperatures (T_e_) were between 102 and 114 °C, indicating different forms of water interaction into the structural rearrangement. These results are in accordance with those reported by Coria et al. [7].

**Figure 5:**
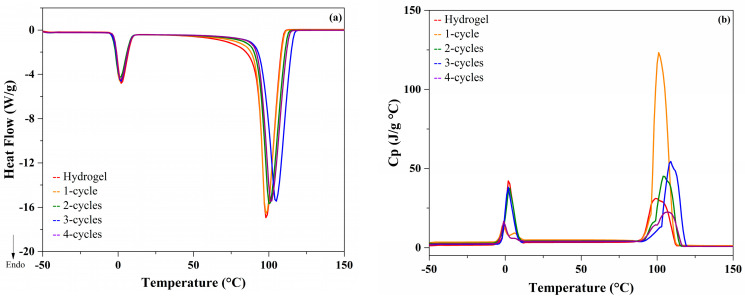
Thermograms of karaya samples (**a**) Heat flow; (**b**) Specific heat (Cp).

To determine changes at the structural level of the xanthan hydro- and cryogels in the main thermal transitions, the Cp was evaluated (Figure 4b). It was found that, unlike that observed in the total heat flow, there were rearrangements at the molecular level in the cryogels that are directly related to the number of freeze–thaw cycles. Both in melting and evaporation, the structural changes were found in the hydrogel due to its disordered structure and, as it undergoes cryostructuring cycles, this value decreased, corroborating the rearrangement of the structure and providing better stability [13,14]. Moreover, the thermograms of the karaya cryogels (Figure 5a) show that during melting there were no significant differences (*p* > 0.05) between the number of freeze–thaw cycles, indicating that both the kinetic and structural events were not altered by the process of rearrangement of the polymeric matrix.

However, the changes in the Cp values during melting (Figure 5b), indicated significant differences (*p* < 0.05), mainly in the karaya cryogels of 1 and 4-cycles. In these samples, the Cp values were lower; consequently, there were no significant changes at the structural level in comparison to xanthan cryogels. In the case of the evaporation zone, more noticeable changes in the heat flow were observed. A displacement in the T_e_ value as a function of the number of freeze–thaw cycles was observed; consequently, it was confirmed that there are structural changes such as kinetics [15].

### 2.3. Structural Order Degree

In the case of xanthan cryogels, the maximum level of structural order was reached with 3-cycles since after this number of events, the structural order decreases, indicating instability of the polymer chains (Table 1). Giannouli and Morris [13] and Nur Hazirah et al. [16] explain that this phenomenon occurs because the ramifications of the xanthan chains bend or protrude at right angles of the main chain, which disorganizes the gel network; consequently, the structural order decreases.

Due to the structure of the karaya gum, the rearrangement levels are lower compared to those of the xanthan samples. This is because their alternating units in the main chain do not allow more orderly interactions to be generated. In this case, the karaya hydrogels have higher values in their molecular arrangement, and, as they experience freeze–thaw cycles, the values decrease. This phenomenon indicates that the cryostructuring process modifies molecular interactions to some extent, meaning that the polymeric matrix does not exist in physicochemical equilibrium, which leads to effects at the structural level.

### 2.4. Activation Energies

*Ea* is defined as the energy required to initiate a reaction [17]. In the case of cryogels (Table 2), *Ea* represents the amount of energy required to start the kinetic process of ice melting. In polymers, it is important to determine this energy, since *Ea* can also be indicative of the degree of order that exists between the polymer chains, which is directly reflected in the width of the vibrational bands observed in FTIR spectroscopy [15,18,19].

In the case of xanthan cryogels, it was found that the lowest *Ea* value corresponds to the structure with the highest degree of ordering, which is directly related to the most homogeneous pore sizes. Thus, the 3-cycle cryogel has the greater physicochemical stability in comparison with the other cryogels. For karaya cryogels, the same trend was observed, indicating that, for the hydrogel, the *Ea* is smaller, which is related to the structural arrangement, size and homogeneity of pore diameters. These results demonstrated that repeated freeze–thaw cycles destabilize the structure of the polymeric matrix, generating weaker and less orderly interactions, which implies that it is not stable enough to control ice crystal formation [17].

### 2.5. FTIR-ATR Studies

**Figure 6:**
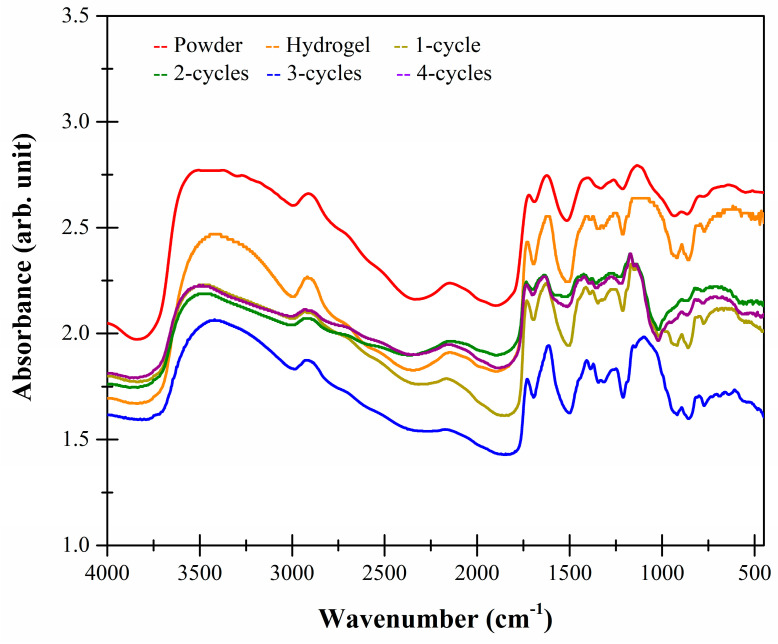
FTIR spectra of xanthan powder, hydro- and cryogels.

Figure 6 shows the FTIR-spectra of the xanthan samples. At 3450 cm^−1^ there were stretching vibrations of the OH^−^ groups associated with the free inter- and intra-molecular groups in the form of glucose dimers and bound water. At 2910 cm^−1^ there was an important band indicating axial stretching of CHO and C–H groups, as well as asymmetric vibrations of CH_2_ methylene groups. In addition, there was a band at 2150 cm^−1^ related to stretching vibrations between C=C=O groups (ketenes), which is characteristic of the polysaccharides.

At 1732 cm^−1^, there were vibrations associated with the carbonyl groups indicating that the salt (COO^−^) and the acid (COOH) forms coexist in the internal mannose chains of the xanthan gum. At 1630 cm^−1^ there were axial flexions of the C=O (diketones), as well as stretching of the esters of methylated galacturonic acid of the polymer. The three-cycle cryogel has a higher band intensity (1630 cm^−1^), indicating that by modifying the structure by freeze–thaw, this type of interaction increases and therefore provides greater stability to the cryogel [20]. Another band appeared at 1415 cm^−1^ related to flexion of the C=O carboxylate and hydroxyl (OH) anions, as well as scissor twisting in the terminal methylenes (CH_2_) of the xanthan chain. It is noted that the cryostructuration process does not generate significant differences (*p* > 0.05) in the intensity of this band. Finally, at 1130 cm^−1^, there were asymmetric stretches of C–O–C aliphatic ether groups, indicating the presence of tertiary C–OH free groups, which do not occur in greater intensity with the 3-cycle cryogel [16,21,22].

**Figure 7:**
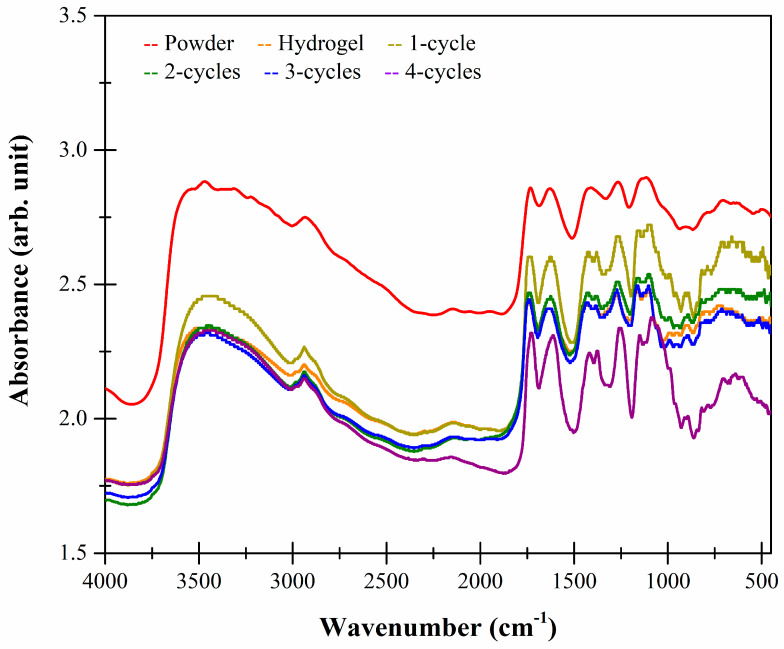
FTIR spectra of karaya powder, hydro- and cryogels.

Figure 7 shows the FTIR spectra of the karaya cryogels. The bands at 3450, 2930 and 2150 cm^−1^ indicate that there were stretching vibrations of the OH^−^ groups associated with the free inter- and intra-molecular groups in the form of glucose dimers, axial stretches of CHO and CH groups, as well as asymmetric vibrations of methylene groups and stretching vibrations between C=C=O groups, respectively.

At 1725 cm^−1^, there were stretching vibrations of the C=O groups of aldehydes and free acetyl groups. The band at 1630 cm^−1^ indicates stretches of the C=O of methylated ester groups of galacturonic acid. These two bands (1725 and 1630 cm^−1^) are characteristic of the karaya gum, which have greater intensity in the 4-cycle cryogel, indicating that the cryogel formation process favors the formation of this type of interaction. Finally, at 1270 cm^−1^, there were asymmetric stretches of C–O–C of aromatic ethers, characteristic of this type of polysaccharide. Lastly, at 1100 cm^−1^, there were stretches of the OH^−^ groups of the secondary alcohols and asymmetric stretching of the pyranose rings [23,24].

### 2.6. Zeta Potential Studies

**Figure 8:**
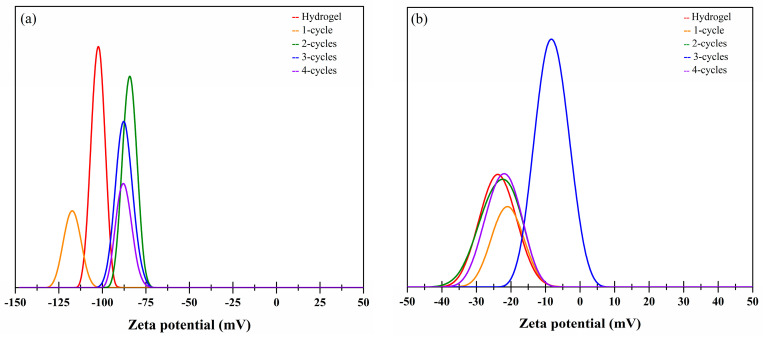
Zeta potential of the hydro- and the cryogels as a function of the freeze–thaw cycles. (**a**) xanthan; (**b**) karaya.

In this research, the zeta potential (ζ) was used to determine the surface charge properties of the hydro- and the cryogels (Figure 8).

In general, xanthan hydrogels are stabilized by repulsive forces, in this sense, steric and electrostatic repulsion are two important repulsive forces [25]. Electrostatic interactions between the same charges in the particles repel each other and consequently higher stability is observed. Generally, if cryogels have higher zeta potential values, the system is considered to be more stable and anti-cohesive, such is the case of the xanthan hydro- and cryogels (Figure 8, profile a). It is well known that karaya gum is a negatively charged biopolymer; therefore, in the hydrogels, a negative zeta potential (ζ) can be expected [26]. However, cryostructuring caused a reduction in this parameter (from −23.48 to −8.07 mV). This means that, despite the values of zeta potential being lower than that observed in the xanthan samples; hydro- and cryogels made from karaya gum were also stable.

### 2.7. Rheological Analysis

**Figure 9:**
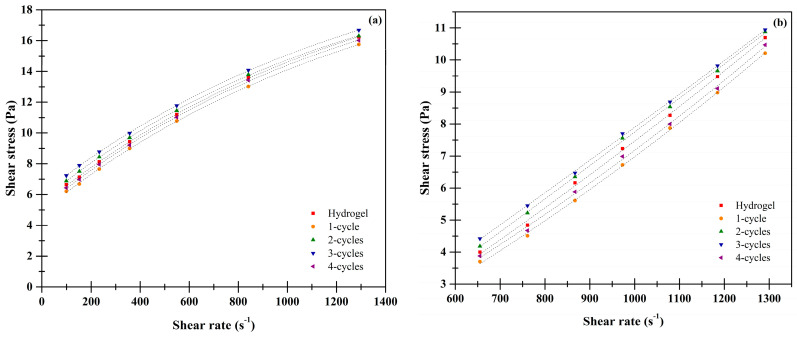
Rheological behavior of the hydro- and cryogels. (**a**) xanthan; (**b**) karaya.

The xanthan and karaya cryogel samples generally behave as non-Newtonian fluids, which conform to the model described by Ostwald de Waele (*Power Law*). The xanthan cryogels had shear thinning behavior (Figure 9a), indicating that the viscosity depends only on factors such as shear rate and temperature as described by Truesdell [6], Tao et al. [20], and Lin et al. [27]. In this sense, the three-cycle cryogel presents its rheological parameters *n* and *k* differently (*p* < 0.05) (Table 3) compared to the other xanthan hydro- and cryogels, denoting that there is a molecular alignment inside the cryogel observed by MDSC and SEM analysis.

In the case of karaya cryogels (Figure 9b), all behaved as a shear thickener fluid, which characterizes this type of material with an increase in viscosity as the shear rate increases, favoring the molecules having a high degree of collisions and, therefore, greater contact between them. This is corroborated with the values obtained from the flow behavior index *n*, where these are greater than 1 [4,28].

## 3. Materials and Methods

### 3.1. Preparation of the Cryogels

Xanthan gum (-C_35_H_49_O_29-n_; Sigma-Aldrich, CAS 11138-66-2, *Xanthomonas campestris*) and karaya gum (C_26_H_34_N_2_O_13-n_; Sigma-Aldrich, CAS 9000-36-6, *Sterculia tree*) were dispersed (0.5% *w/w*) with magnetic stirring in distilled deionized water at 80 °C for 2 h, adapting the methodology suggested by Giannouli and Morris [13] to form hydrogels. Freezing was carried out using the protocol described by Coria et al. [7] to form the cryogels. Briefly, 50 mL of the dispersions were frozen by indirect contact with liquid nitrogen up to −150 ± 1 °C for 10 min and thawed by immersion in water at 50 ± 2 °C for 30 min until completing four freezing–thawing cycles.

### 3.2. Scanning Electron Microscopy (SEM)

The surface morphology and the microstructure of the cryogels were examined with a JSM-6010LA SEM microscope (Jeol, Tokyo, Japan). Briefly, samples were freeze dried in a Labconco Freezone 4.5 L Benchtop Freeze–Dry System. Lyophilization was carried out for 24 h at −49 °C under a chamber vacuum pressure of 3.5 Pa. For microscopy, samples were coated with a gold layer at 10 mA for 5 min using a Denton Desk V ion sprayer (Denton Vacuum, NJ, USA). The analysis was performed at 900 × with an accelerating voltage of 12.5 kV at a high vacuum. The dimensions of the pores were determined with the software JMicroVision version 1.2.7 [7].

### 3.3. Modulated Differential Scanning Calorimetry (MDSC) Analysis

Samples (4.5 ± 0.1 mg) were analyzed using a differential scanning calorimeter 2920 series with temperature modulation (TA Instruments, New Castle DE, USA). The temperature calibration was carried out with indium (156.6 °C) and the heat capacity with sapphire (aluminum oxide). Nitrogen was used as a purge gas at a constant flow of 60 mL/min in the refrigerated cooling system (RCS) to avoid condensation inside the cell. Samples were equilibrated at −50 °C and heated in the modulated DSC furnace at a rate of 5 °C/min with temperature modulation of 0.8 °C every 60 s [15].

### 3.4. Structural Order Degree

The structural order degree was determined following the recommendations by Patel et al. [29] using the enthalpy of fusion of the polymers and the following equation:

(1) $$X_{c}=\frac{\Delta H_{f}}{\Delta H_{f}^{0}}$$

where *X_c_* is the ordered fraction of the polymer, $\Delta H_{f}$ is the fusion enthalpy of the polymer dispersion (J/g) and $\Delta H_{f}^{0}$ is the fusion enthalpy of the crystalline polymer (J/g).

### 3.5. Activation Energies (Ea)

The *Ea* values were obtained using the methodology described by Coria et al. [15]. The reaction order (*n*), the Arrhenius constant (*Z*), the degree of conversion (α), and the conversion rate $\left(\frac{d\alpha }{\mathrm{dt}}\right)$ were determined using the following expressions:

(2) $$\ln\left(\frac{d\alpha }{\mathrm{dt}}\right)=\ln Z-n\ln\left(1-\alpha \right)-\frac{Ea}{\mathrm{RT}}$$

(3) $$\alpha =\frac{\Delta H_{g}}{\Delta H_{t}}$$

where ΔH_g_ is the enthalpy for each temperature in the transition zone (J/g), and ΔH_t_ is the total enthalpy (J/g). To obtain the value of the unknown factors (*Z*, *n* and *Ea*), a multiple linear regression (MLR) analysis of Equation (2) was performed [25,26].

### 3.6. Fourier Transform Infrared Spectroscopy with Attenuated Total Reflection (FTIR-ATR) Studies

The functional groups were characterized using a FTIR Frontier SP8000 dual spectrophotometer (Perkin Elmer, MA, USA) equipped with a deuterated triglycine sulfate detector (DTGS) and controlled with Spectrum 10.4.2 software (Perkin Elmer Ltd., Bucks, UK). The powdered samples (<250 µm) were placed in the ATR accessory and analyzed from 4000 to 450 cm^−1^ at a resolution of 4 cm^−1^ by co-adding 32 scans. Spectrums were collected in quadruplicate in absorbance mode [7].

### 3.7. Zeta Potential (ζ)

Zeta potential measurements were carried out using the ZetaSizer Pro (Malvern Instruments, Worcestershire, UK) at 25 °C using the DTS1070 cell and an equilibrium time of 15 s following the recommendations of Pérez-De León et al. [30].

### 3.8. Rheological Analysis

The rheological properties of the hydro- and cryogels were measured by using a rotational viscosimeter (Rheomat RM180, Mapple Instruments, Toronto, ON, Canada) with attached computer software (RSI Orchestrator, Rheometrics Scientific, Piscataway, NJ, USA). A total of 25 mL of each sample were kept in a thermostatically controlled water bath for 10 min before being measured in order to attain a desirable temperature of 25 °C. Measurements were taken 2 min after the spindle was immersed in each sample, in order to allow thermal equilibrium in the sample. The shear rate versus shear stress data were interpreted using the power law expression (τ = k⋅γ^n^); where τ is the shear stress (Pa), γ is the shear rate (s^−1^), *n* is the flow behavior index, and *k* is the consistency index (Pa⋅s^n^).

### 3.9. Experimental Design and Statistical Analysis

The experiment was carried out as a completely randomized design with three replicates. Mean, standard deviation, one and 2-way ANOVA and comparison of means by the Tukey test were performed using the Minitab 16.0.1 software (Penn State University, Pennsylvania, USA). A significance value of *p* < 0.05 was utilized to identify significant differences between treatments.

## 4. Conclusions

Xanthan gum when subjected to three freeze–thaw cycles alters the orientation of its structure, favoring thermal stability, as well as the formation of new bonds within the polymeric matrix, decreasing pore size, modifying Cp values, decreasing *Ea* values, and providing a greater degree of structural order and fluidity.

In the case of karaya hydrogel, it was found that although it was a relatively stable polymer at low temperatures, the freeze–thaw process destabilized its structure, causing non-homogeneity in pore sizes, and modifying its thermal properties; consequently, karaya could not be considered as a good cryogel based on its structural, molecular, rheological and thermal properties.

Taking these results together, the 3-cycle xanthan cryogel, has broad prospects for future study with potential applications in food preservation processes, including freezing, controlling the growth of ice crystals, thawing while avoiding losses of exudates, and high-temperature treatments such as pasteurization, sterilization, drying, and others. It can also be used to assist emerging technologies such as ultrasound or even in the application as a coating or biofilm.

## Figures and Tables

**Table 1:** Structural order percentage of the hydro- and cryogels.

Sample	Xanthan	Karaya
Hydrogel	93.43 ± 0.48 ^a^	87.16 ± 0.68 ^c^
One cycle	92.34 ± 0.55 ^a^	79.89 ± 1.21 ^a^
Two cycles	94.36 ± 0.71 ^b^	80.40 ± 1.38 ^a^
Three cycles	95.55 ± 0.92 ^c^	84.48 ± 1.74 ^b^
Four cycles	92.17 ± 0.34 ^a^	78.28 ± 1.91 ^a^

Mean ± Standard deviation; Means with different letters (*a*, *b* and *c*) in the same column, are statistically different (*p* < 0.05).

**Table 2:** Activation energies (kJ/mol) of the hydro- and cryogel samples.

Sample	Xantana	Karaya
Hydrogel	196.48 ± 1.24 ^b^	174.83 ± 0.99 ^a^
One cycle	250.28 ± 1.12 ^d^	376.12 ± 1.85 ^c^
Two cycles	200.74 ± 1.57 ^b^	388.10 ± 1.33 ^c^
Three cycles	154.79 ± 0.95 ^a^	244.17 ± 1.52 ^b^
Four cycles	212.12 ± 1.03 ^bc^	377.90 ± 1.74 ^c^

Mean ± Standard deviation; Means with different letters (*a*, *b* and *c*) in the same column, are statistically different (*p* < 0.05).

**Table 3:** Rheological parameters according to the power’s law.

Sample	Xanthan		Karaya	
	*n*	*k*	*n*	*k*
Hydrogel	0.3580 ± 0.0002 ^b^	1.2001 ± 0.0010 ^a^	1.3375 ± 0.0031 ^a^	0.0008 ± 0.0003 ^b^
One cycle	0.3728 ± 0.0024 ^c^	1.0466 ± 0.0009 ^a^	1.4048 ± 0.0052 ^b^	0.0005 ± 0.0001 ^b^
Two cycles	0.3427 ± 0.0010 ^a^	1.3496 ± 0.0011 ^b^	1.4646 ± 0.0033 ^c^	0.0003 ± 0.0001 ^a^
Three cycles	0.3303 ± 0.0021 ^a^	1.5022 ± 0.0024 ^c^	1.4724 ± 0.0049 ^c^	0.0003 ± 0.0000 ^a^
Four cycles	0.3645 ± 0.0028 ^bc^	1.1322 ± 0.0008 ^a^	1.5163 ± 0.0041 ^d^	0.0002 ± 0.0000 ^a^

Mean ± Standard deviation. Means with different letters (*a*, *b* and *c*) in the same column, are statistically different (*p* < 0.05).

## Data Availability

Not applicable.

## Sample Availability

Samples of the compounds are not available from the authors.
